# The giant steps in surgical downsizing toward a personalized treatment of vulvar cancer

**DOI:** 10.1111/jog.15103

**Published:** 2021-12-28

**Authors:** Andrea Giannini, Ottavia D'Oria, Benito Chiofalo, Valentina Bruno, Ermelinda Baiocco, Emanuela Mancini, Rosanna Mancari, Cristina Vincenzoni, Giuseppe Cutillo, Enrico Vizza

**Affiliations:** ^1^ Gynecologic Oncology Unit, Department of Experimental Clinical Oncology IRCSS‐Regina Elena National Cancer Unit Institute Rome Italy; ^2^ Department of Medical and Surgical Sciences and Translational Medicine, PhD Course in “Translational Medicine and Oncology” Sapienza University Rome Italy

**Keywords:** inguinofemoral lymphadenectomy, radical vulvectomy, sentinel lymph node biopsy, Taylor therapy, vulvar cancer

## Abstract

The present article aims to highlight the importance of changes of personalized surgical treatment for vulvar cancer. Current international literature regarding surgical treatment of vulvar cancer was evaluated. This included several studies and systematic reviews. Radical surgery approach, such as en bloc resection, was the first therapeutic option and the standard care for many years, even if burdened with a high complication rate and frequently disfiguring. Taussing and Way introduced radical vulvectomy approach with en bloc bilateral inguinal‐femoral lymphadenectomy; modified radical vulvectomy was developed, with a wide radical excision of the primary tumor. The role of inguinofemoral lymphadenectomy (mono or bilateral) changed in the years too, particularly with the advent of SLN biopsy as minimally invasive surgical approach for lymph node staging, in patients with unifocal cancer <4 cm, without suspicious groin nodes. More personalized and conservative surgical approach, consisting of wide local or wide radical excisions, is necessary to reduce complications as lymphedema or sexual disfunction. The optimal surgical management of vulvar cancer needs to consider dimensions, staging, depth of invasion, presence of carcinoma at the surgical margins of resection and grading, with the goal of making the treatment as individualized as possible.

## Background

Vulvar cancer (VC) accounts for 5% of all gynecologic cancer, usually affecting patients aged over 65 years.^1^, ^2^ In the past decades, the incidence of VC in young women is alarming rising.^3^ Squamous cell carcinoma is the most common histological type (up to 90%).^4^ Human papilloma virus (HPV)‐related dysplasia is typical of younger women; in older patients, there is a connection with vulvar dermatoses, such as lichen sclerosis.^5^, ^6^ The clinical presentation includes a visible or self‐palpated lesion, frequently with pruritus, discharge, or bleeding.^7^

The staging of vulvar cancer is surgical, based on the 2009 Federation International de Gynecology et Obstetrique (FIGO) and American Joint Committee on Cancer (AJCC) Seventh Staging Edition TNM staging. Vulvar biopsy is mandatory to assess stroll invasion; clinical and radiologic assessment of tumor dimension is mandatory too; moreover, surgical and/or radiological assessment of pelvic lymph node spread and distant metastasis is necessary.^8^, ^9^

The management of VC depends on disease stage. Surgical approach is determinate by tumor size and location, histologic and cytologic grade, depth of invasion, vascular space invasion and, particularly, nodal metastasis that represents the most important prognostic factor.^10^, ^11^ For early‐stage disease, a pelvic magnetic resonance imaging (MRI) could be useful to define tumor dimension and locoregional disease spread, whereas for advanced‐stage disease, a whole‐body computed tomography (CT) scan or a whole‐body positron emission tomography (PET)/CT scan should be considered for an accurate evaluation.^12^, ^13^ Moreover, every patient needs complete blood count, infectious screening, renal and hepatic function tests; a physical examination with cervical pap smear is mandatory too.^14^

The identification of new molecular markers for prognostic purposes is needed. Epidermal growth factor receptor (EGFR) immunohistochemical overexpression/gene amplification and p53 overexpression have been correlated with a worse prognosis. Programmed death ligand PDL‐1 seems to be a useful target for new therapeutic approach. The positivity to certain molecular markers does not influence the surgical treatment.^15^

## Results: Surgical Treatment

Over the last years, the approach to VC treatment has evolved from invasive surgery to more conservative approaches, becoming as personalized as possible, with the integration of new surgical techniques. In addition, the radical removal of the tumor can be achieved through a more tissue‐sparing vulvar surgery.^16^, ^17^

### Early‐stage vulvar cancer

#### Surgical management

**FIGURE 1:**
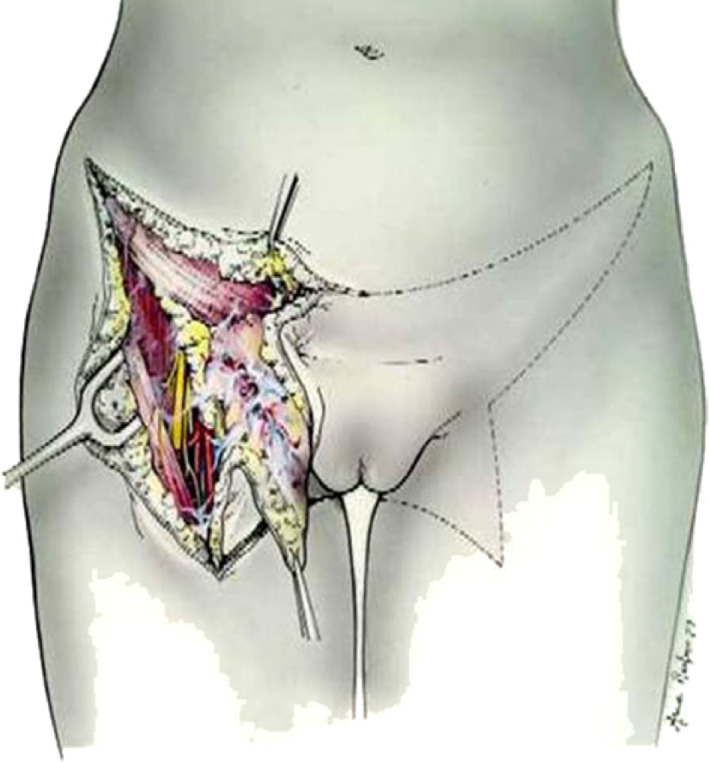
En bloc Way–Taussing radical vulvectomy showing butterfly skin incision^19^

**FIGURE 2:**
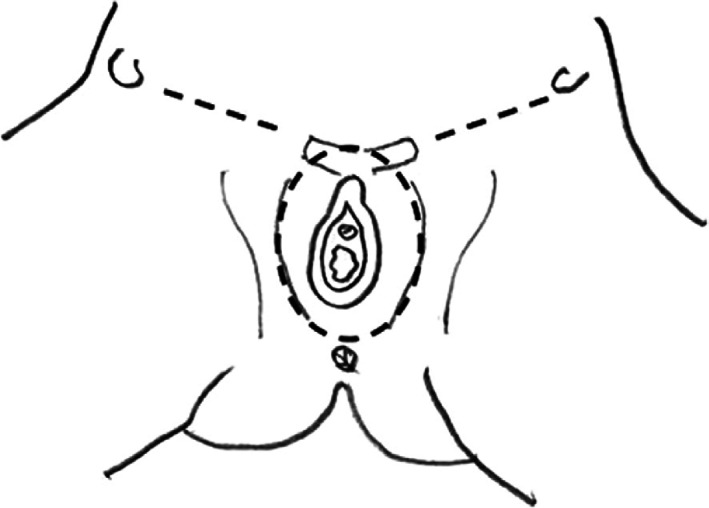
Triple incision: A skin bridge is left between the vulval and the groin incisions^19^

Early‐stage VC includes FIGO Stages I and II, with tumor size ≤4 cm and stromal invasion ≤1 mm. Nodal spread is absent. Stages IA, IB, and II ≤4 cm are treated surgically. For tumors >1 mm invasion and dimensions up to 4 cm, surgical approach consists in a modified radical vulvectomy, with surgical lymph node assessment. This surgical technique includes superficial and deep fascia lata, including separate incisions for tumor and groin node dissection^18^; in this way, radical vulvectomy approach with en bloc bilateral inguinal‐femoral lymphadenectomy has been overcome, sparing several complications (Figures 1 and 2). In fact, the postoperative management of the traditional surgical approach was very difficult because of the onset of many complications and surgical sequelae (infection, necrosis, pain, functional and esthetic distortion, deterioration of sexual life and psychological health)^14^ Di Saia and Hacker developed the concept of minimal resections margins, limited to the tumor.^20^, ^21^, ^22^, ^23^ These results have been confirmed by a large study conducted by the Gynecologic Oncology Group.^24^ Safe margins are considered and are maintained from 1 to 2 cm (according to Heaps' study).^25^ The resection of primary vulvar tumor aims to save organs, such as the urethra, clitoris, and anal sphincter, while maintaining an adequate surgical radicality for the patient; the site of incision depends on tumor location.^18^, ^21^ For substage VC IA ≤1 mm treatment consists of a wide local excision, adequate if margins are negative. The term “wide local excision” or “simple vulvectomy” (synonymous of wide local excision) is referred to a type of excision without the inclusion of deep fascia but limited to subcutaneous tissue; tumor margin is 1 or 2 cm above the primary vulvar tumor.^20^, ^21^

There are situations where close margins are more common (proximity to the clitoris, urethra, or vulva), but the National Comprehensive Cancer Network (NCCN) Guidelines recommend re‐excision of positive margins or those classified as close (<8 mm).^26^ If smaller margins are safe is subject of studies.^27^

Moreover, postoperative reconstruction, based on patients' characteristics, after demolitive surgeries has improved esthetic result and psychological acceptance of this pathology, representing an important step for personalized treatment. Two types of flaps were identified: Advancement Flap (V‐Y Gluteal Fold Flap; Medial Thigh Flap) and Transpositional Flap (Lotus Petal Flap; Gluteal Thigh Flap; Gluteal Fold Flap and Anterolateral Thigh Flap).^28^, ^29^

#### Resection margins

The safety of the size of resection margin is debated. Non‐pathological margins must be greater than 8 mm^25^ Chan et al. suggested that no local recurrence has been registered after at least 8 mm margins distant.^30^ The study of Woelber showed that the recurrence rate is the same for lesions with margins of less than 8 mm and at least 8 mm, demonstrating no impact of margins distance on progression free survival (PFS).^31^ Arvas et al., assessing the margin status in 61 patients affected by vulvar cancer, analyzed those women with pathological margins ≤2 mm had an high risk of recurrence, compared with the group with >2 mm. The intermediate margins value (2–8 mm) was not a predictor of local recurrence.^32^, ^33^ The use of re‐excision or adjuvant radiotherapy on the basis of close surgical margins alone (2–8 mm) should be carefully considered.^27^ Höckel et al. proposed a novel approach for patients with vulvar cancer based on compartmental tumor spread and based on ontogenetic anatomy: in this prospective trial patients were treated with vulvar field resection and anatomical reconstruction, considering anatomy from embryonic development. The extent of deep vulvar resection is not defined with conventional surgical margins and this approach allows to preserve tissue for esthetic reconstruction.^23^

However, current recommendations suggest surgical margins of 2 cm and final pathological margin of at least 1 cm.

#### Sentinel lymph node (SLN) and groin treatment

Surgical assessment of nodes can be achieved with bilateral SLN biopsy or inguinofemoral lymphadenectomy [IFLND]). Node's evaluation is necessary because the risk of occult nodal metastases is up to 30%.^34^ Utilization of SLN represents one of the biggest steps for surgical treatment of vulvar cancer, avoiding complications of routine bilateral lymphadenectomy (risk for lower‐extremity lymphedema (approximately 30%–70%).^35^, ^36^, ^37^, ^38^, ^39^ This routine approach was changed by Gynecologic Oncology Group (GOG) study in 1987, avoiding groin node dissection in microinvasive VC, with a low risk of nodal metastases and in 1993^36^ Homesley assessed that VC localized >2 cm from the midline, drains to ipsilateral groin nodes, and did not metastasize to contralateral part; in this way bilateral groin dissection became not mandatory. The advent of SLN biopsy provides new opportunities for patients, reducing lymphedema or lymphocists, out increasing the risk of groin recurrence.^40^, ^41^

SLN is the first lymph node that drains from tumor; GOG 173 and GROINSS‐V–1 were the two multicenter observational studies that have analyzed the safety and feasibility of SLN as valid alternative to IFLND.^35^, ^42^ For midline vulvar tumors, bilateral SLN should be performed; whereas for lesions that are located ≥2 cm from the midline, unilateral node dissection is sufficient.^20^ Currently SLN biopsy has become the standard care for surgical treatment of VC with size ≤4 cm and clinically and/or radiological negative inguinofemoral lymph node. In case of positive SLN, the postoperative management is debated: alternatives include completion lymphadenectomy or external beam radiation therapy (EBRT). The ongoing prospective trial (GOG 270/Groningen International Study on Sentinel Nodes in Vulvar Cancer (GROINSS‐V‐II) is evaluating if radiation therapy is safe in patients with SLN micrometastes (Table 1).^43^, ^44^, ^45^, ^46^, ^47^, ^48^, ^49^

**TABLE 1:** Literature review of the use of sentinel lymph nodes biopsy in vulvar cancer

Authors	Years	Study type	Patients (groins)	Mapping method	Median FU	Groin recurrence (%)	Outcome in SLN negative patients (%, 95% CI)
van der Zee (GROINSS‐V study)^37^	2008	Prospective	403 (623)	R + B	35 (2–87)	6/259 (2.3) unifocal disease; 8/276 (3) including multifocal disease	3‐year DSS (97)
Oonk^42^	2010	Prospective	403	R + B	120	11 (2.7)	NA
Levenback (GOG 173)^41^	2012	Prospective	452 (772)	R + B	NA	NA	NA
Woelber^44^	2013	Retrospective	Primary SLN group = 74/106 Secondary SLN group = 32/106	R	33 (3–118)	Primary SLN group = 4/74 (5.4); Secondary SLN group = 0	Primary SLN group = 3‐year DFS (72.5) Secondary SLN group = 3‐year DFS (92.5)
Robison^45^	2014	Prospective	86	R + B	58	4/86 (4.7)	NA
Te Grootenhuis^46^	2015	Prospective	377	R + B	105 (0–179)	6/253 (2.5) unifocal disease	5‐year DSS (93.5) 10‐year DSS (90.8) 5‐year OS
Klapdor^47^	2017	Retrospective	772	R or B	33 (0–156)	2/69 (2.9)	3‐year PFS (82.7; 72.3–92.7) 3‐year OS (92.7; 85.7–99.7)
Nica^48^	2019	Retrospective	159 (245)	R or R + B	31	6/120 (5)	1‐year PFS (90) 5‐year PFS (80)

Abbreviations: B, blue dye; DFS, disease‐free survival; DSS, disease‐specific survival; OS, overall survival; PFS, progression‐free survival; R, radiotracer.

For women with diagnosis of vulvar cancer, the presence of lymph node metastases is the most important prognostic factor.^50^ The radical lymph node (LND) dissection was used for years, although a very high morbidity (lymphedema, nerve injury) with compromised quality of life.^51^ Moreover, histological analysis confirms the presence of lymph node metastases only in the 25%–35% of all patients; in this way the benefits from the LND procedure were limited SLN dissection as valid alternative to LND has been proposed to avoid overtreatment and to control complications. GROINSS‐V is a prospective multicentric study: 400 patients with the same tumoral characteristics (size, stromal invasion, and negative preoperative diagnostic assessment) were treated with sentinel procedure. In patients with negative biopsy, systematic lymphadenectomy was omitted. Groin recurrence rate was only 2% after almost 3 years. No significative differences with patients with early‐stage vulvar cancer treated with groin lymphadenectomy were noted.^37^ The number of groin recurrence in sentinel‐node negative patients seems to be comparable to the other reported for early‐stage vulvar cancer treated with lymphadenectomy. So, the effect seems to be the same.^52^

Oonk et al. demonstrated from the GROINSS‐V data that even when only isolated cells are found in the sentinel node, the rate of no sentinel node metastasis is 4.1%, and in cases of metastasis of less than 5 mm, 11.7%.^42^, ^43^

GOG 173 is a prospective study in early‐stage vulvar cancer, in which patients with SLN mapping followed by standard complete IFLND. The false‐negative rate of an SLN biopsy in GOG 173 was 2.7% in patients whose tumors were <4 cm.^41^ Thanks to results of these studies, SLN was considered safe, sparing serious complications.

A systematic review and meta‐analysis of the cumulative data on SLN detection reported a per‐groin detection rate of 87% and a false‐negative rate of 6.4% and groin recurrence rates appeared to be similar only under optimal conditions (unifocal tumors <4 cm, clinically non‐suspicious nodes in the groin, appropriate techniques, and procedures).^53^

Recent studies checked safety and feasibility of sentinel node biopsy after vulvar surgery, confirming that this procedure after previous surgery is safe and reflects groin status.^42^, ^54^, ^55^ However false‐negative sentinel carries a high risk of mostly fatal groin recurrences. Particularly midline tumors larger than 2 cm have to be treated carefully, because they are mostly found in cases with groin recurrences after sole SLN.^56^

In conclusion, patients with unifocal vulvar cancer, tumor size less than 4 cm, and clinically negative groin assessment can undergo SLN and vulvar surgery in a center with experienced team; if the sentinel node biopsy is positive, patient should undergo systematic IFLND. However, the optimal postoperative management of positive SLN is debated; in fact, adjuvant radiotherapy seems to be a valid alternative. The results of GROINSS‐V‐II trial show that for positive SLN with metastasis ≤2 mm radiotherapy is a valid therapeutic option instead of IFLND; toxicity is minimal. For patients with positive SLN and metastasis >2 mm, radiotherapy does not seem to be a safe alternative but systematic IFLND is the best option.^42^

The current standard approach for detection of SLN includes the use of lymphoscintigraphy with technetium 99 m with intraoperative blue dye (methylene blue or indigo carmine), whereas the use of blue dye alone is not recommended.^53^

### Management of locally advanced vulvar cancer

For women affected by VC, with unresectable disease, treatment of choice consists of radiotherapy (RT) combined with chemotherapy, usually cisplatin. Radical resection in the past was the standard care for the treatment of locally advanced VC; GOG 101 demonstrated that only 3% of patients with T3 and T4 tumors had residual unresectable tumors following chemoradiation.^57^

Particularly, in tumors with negative node metastasis RT limited to the vulvar tumor alone can be sufficient; instead, it is necessary to involve the pelvis and groin in case of positive lymph nodes. In cases with groin nodes involvement, surgery would be the best choice, but RT is a valid alternative for fragile patients not eligible for surgery. Clinically suspicious nodes need to be confirmed by biopsy; if there is no radiographic or clinical evidence of nodal metastases, groin nodes should be evaluated by IFLND, because of the risk of false‐negative.^44^

For patients with Stage IIIB, IIIC, and IVA, chemoradiation to the vulvar tumor, groin, and pelvis is the gold standard. Additional surgery after this approach can be considered in cases of residual disease. Total pelvic exenteration is reserved for selected patients. In fact, this approach is an option for patients with involving of urethra, anus or vagina, and other organs. Surgical morbidity is high with median survival of 11 months.^58^ A recent study by MD Anderson Cancer Center included reported a 5‐year overall survival rate of 22%.^59^ Women who have no other viable alternatives can benefit from this approach.

### Management of recurrence

Recurrent disease occurs in 15%–35% of women with VC. Surgery can be an adequate treatment for recurrent disease limited to the vulvar area, with a cure rate up to 80%; the incidence of isolated local recurrence is 20%. The type of surgery is based on the location and dimensions of the recurrence (wide local excision, hemivulvectomy, or radical vulvectomy).^58^, ^60^ Different studies focused on exclusive surgical approach for local recurrence, with a rate of second recurrent of 25%–50%.^61^

The management of groin recurrence is debated and difficult because patients die of recurrence. Surgery, followed by radiotherapy, is currently the treatment of choice. Surgery (IFLND or debulking surgery of groin recurrence), either alone or in combination with radiotherapy, has been investigated and patients with combined therapy (surgery and chemoradiotherapy) had a better overall survival.^62^

Decision about the best treatment choice mainly depends on location of recurrence, performance status of patient, previous treatment, resulting in a tailor‐made approach.

## Discussion

Surgical treatment of VC has changed in the last years. The standard mutilating radical vulvectomy has evolved, promoting a conservative and personalized approach. The approach to groin surgery is deeply changed too.

Wide local excision and modified vulvectomy are surgical options that preserve women' s quality of life, reducing side effects like lymphedema, sexual dysfunction, urinary complications, and psychological compromission. No randomized clinical trial has been conducted to compare wide local excision to radical vulvectomy. Oncologic safety seems to be equal.^63^ Patients with early stage unifocal squamous cell cancer of the vulva (<4 cm) and no suspicious and/or enlarged lymph nodes at imaging should be considered for SLN biopsy.^52^

In recent years, quality of life of patients undergoing surgery for vulvar cancer has become a central topic in different studies, particularly risk of lymphedema, causing discomfort heaviness and reduced mobility. A prospective trial by GOG^32^ demonstrated that the incidence of lower limb lymphedema is 65% at 6 months after IFLND. On the contrary, in case of SLN this rate is 2%.^34^

The objective of GOG study 244 is to evaluate the incidence and risk factors for lymphedema associated with surgery for gynecologic malignancies, but there were too few VC patients for certain results, therefore with lack of exhaustive results.^64^

In conclusion, surgery is the primary treatment of vulvar cancer. Early‐stage disease has a very good prognosis and treatment should be individualized. The procedure should only be performed by an experienced multidisciplinary team, and in well‐selected patients.

Individualization of surgical treatment makes it possible to improve the quality of life and psychological state of these women, without sacrificing security and safety.

## Conflict of Interest

The authors declare that there is no conflict of interest.

## Author Contributions

Conceptualization: Andrea Giannini and Ottavia D'Oria; supervision: Giuseppe Cutillo and Enrico Vizza; writing—original draft preparation: Andrea Giannini, Ottavia D'Oria, Benito Chiofalo, and Ermelinda Baiocco; review and editing: Rosanna Mancari, Cristina Vincenzoni, and Emanuela Mancini. All authors have read and agreed to the published version of the manuscript.

## Data Availability

No data are available.
